# In Ovo Injection of Dietary Supplement: Effect on Egg Weight, Embryonic Development, Hatching, and Chick Quality Traits in Broiler Chickens

**DOI:** 10.1002/vms3.71107

**Published:** 2026-07-21

**Authors:** Kadriye Kursun, Nasir Abdallah, Mikail Baylan

**Affiliations:** ^1^ Faculty of Agriculture, Department of Animal Science Cukurova University Saricam Türkiye

**Keywords:** broiler, chick quality, embryogenesis, in ovo injection, nutrition

## Abstract

**Background:**

Restricting nutrients in eggs may hinder the growth of commercial chickens, which could result in higher embryonic mortality and poor growth performance, indicating the significance of in ovo injection of dietary nutrients to support embryonic and post‐hatch growth in poultry.

**Objective:**

This study investigated the in ovo supplementation of a dietary supplement on egg weight, embryonic development, hatching, and chick quality traits of broiler chickens.

**Method:**

A total of 300 hatching eggs were randomly pre‐assigned to a specific in ovo injection protocol (T‐1, T‐2, T3, and T‐4) and incubated under standard conditions. At embryonic day (ED) 12, the eggs were either injected with distilled water or a dietary supplement (containing a mixture of vitamins, trace minerals, and amino acids). The injection treatment consisted of T‐1 (non‐injected eggs), T‐2 (eggs injected with 5 mL of distilled water), T‐3 (eggs injected with 5 mL of a solution containing 0.04% of the dietary supplement), and T‐4 (eggs injected with 5 mL of a solution containing 0.08% of the dietary supplement).

**Results:**

The results revealed that the experimental treatment had no effect on egg weight or egg weight loss during embryogenesis (*p* > 0.05). The lowest weight of yolk‐free body mass (YFBM‐w), yield of yolk‐free body mass (YFBM‐Y), embryo length (Em‐L), embryo width (Em‐W), tibia length (TL), and wing length (WL) at ED 16 were identified in T‐4 (*p* < 0.05). At ED 19, the highest Em‐L and eye width (Ey‐w) were identified in T‐3 (*p* < 0.05). The highest chick weight at hatch (CWAH) and chick yield (CY) were identified in T‐4 (*p* < 0.05). While the chick length and appearance score were lowest in T‐4, the chick eye score was lowest in T‐2 (*p* < 0.05). The embryonic mortality, hatchability, navel, and leg scores of chicks were similar among the treatments (*p* > 0.05).

**Conclusion:**

It was concluded that the in ovo injection of a mixture of dietary nutrients could improve embryonic traits during the latter part of embryogenesis, chick weight, and chick yield at hatch; however, it may possess a strong negative effect on hatchability, embryonic mortality, and chick quality traits.

## 1 Introduction

The rearing or production of layer chickens for eggs and broiler chickens for meat has increased exponentially due to the global increase in the consumption and demand of chicken eggs (Abdallah et al. 2022; Abdallah et al. 2024a) and meat. The higher demand and consumption of chicken meat and eggs are mostly due to the lack of any religious condemnation and their affordability (Baylan et al. 2024; Kursun et al. 2024a; Kurşun et al. 2024b). In addition, it was reported (Pomaah et al. 2023) that the demand for poultry products, especially eggs, has increased tremendously due to increasing levels of income and standard of living. To ensure the regular supply of chicken meat and eggs, breeder companies have increased their capacities, increasing the number of hatching eggs produced daily, monthly, and annually.

Additionally, genetic selection for improved feed efficiency, growth, and breast meat yield has also influenced embryonic growth and development in chickens (Tona et al. 2004; Druyan 2010; Nangsuay et al. 2015). Indeed, Ohta et al. (2004) reported that between embryonic day (ED) 11 and 19, the embryos of commercial broiler chickens were heavier than those of commercial layer chickens. The faster embryonic development in commercial broilers and layers has increased embryonic heat production, as well as the requirement for nutrients and energy, for embryonic and early post‐hatch growth and development. However, eggs only supply a small amount of vital nutrients and energy, and this has a detrimental effect on embryonic growth and early post‐hatch development. Indeed, Akosile et al. (2023) reported that restricting nutrients in eggs may hinder the growth of commercial chickens, which could result in higher embryonic mortality and poor growth performance. Foye et al. (2006) also reported that the amount of total protein in eggs emerges as a crucial factor for embryonic growth; however, eggs of broiler breeders have been reported to be rich in fat with a relatively low protein content (Ohta et al. 1999). Moreover, the primary cause of embryonic mortality between ED11 and ED14 has been associated with the scarcity of nutrients (Tona et al. 2022), indicating the significance of amino acid supplementation during ED14 (Uni and Ferket 2004).

Trace minerals (Zn, Fe, Na, Ca, and Mg) are also essential for embryonic growth and development due to their role in the formation and calcification of the skeleton. For instance, due to their involvement in numerous enzymatic reactions and regulation of numerous cellular processes, such as DNA synthesis, red blood cell production, oxygen transport, DNA metabolism, cellular energy production, cell proliferation, apoptosis, organ, and embryonic development, zinc and iron have been reported to be essential for embryonic growth and development (Beyersmann and Haase 2001; Luckheeram et al. 2012; Costa et al. 2023). Vitamins (A, B, C, D, and E), on the other hand, have been reported to decrease late embryonic mortality; improve hatchability, antioxidant status, embryo survivability, and nervous system development; regulate calcium metabolism, which is essential for embryonic skeletal development; reduce embryonic malformation; and improve chick quality and chick weight (Araújo et al. 2019; Kalaba et al. 2024).

Therefore, to supply and increase the availability of nutrients for embryonic growth and post‐hatch development of chickens, the practice of in ovo feeding has been deemed a useful strategy (Foye et al. 2006). This process (in ovo) involves the provision of developing embryos with bioactive substances such as minerals, amino acids, prebiotics, probiotics, symbiotic, creatine, and growth hormones usually between ED 12 and ED 20 (Jadhav 2025). Indeed, previous studies have shown that in ovo feeding improves post‐hatch growth, feed conservation rate, and disease resistance by stimulating embryonic metabolic capacity and gastrointestinal development (Uni and Ferket 2004; Macalintal 2012; Lee et al. 2014; El‐Deep et al. 2020).

Research has investigated the effect of in ovo injection of amino acids, vitamins, and minerals separately. To our knowledge, this is the first study to examine the in ovo injection of a single dietary supplement containing all the essential nutrients (amino acids, vitamins, and minerals) and its effect on detailed aspects of embryonic development, hatching traits, and chick quality in broiler chickens.

## 2 Materials and Methods

### 2.1 Animal Material and Experimental Treatments

A total of 300 hatching eggs of broiler breeders at 29 weeks of age (wk) were used in this study. The eggs were first weighed using a scale of 0.1 g precision, randomly pre‐assigned to a specific in ovo injection protocol (T‐1, T‐2, T3, and T‐4), and incubated under standard conditions (37.8 oC and 65% humidity). At embryonic day (ED) 12, the eggs were weighed again, and the application of the in ovo injection protocol was practised. The injection protocol/treatment consisted of T‐1 (non‐injected eggs), T‐2 (eggs injected with 5 mL of distilled water), T‐3 (eggs injected with 5 mL of a solution containing 0.04% of the dietary supplements), and T‐4 (eggs injected with 5 mL of a solution containing 0.08% of the dietary supplements). The dietary supplement used in the current study was purchased from a certified Turkish company, and the ingredients in the supplement are given in Table 1. In addition, the injection was carried out using a syringe, targeting the amniotic sac as indicated by Tayam and Karadaş (2025).

**TABLE 1:** The nutritional composition of the dietary supplement used in the present study.

Nutritional composition	Unit
Vitamin A	20,000,000 IU
Vitamin D3	2,000,000 IU
Vitamin E	8000 mg
Vitamin B1	3000 mg
Vitamin B2	2400 mg
Niacin	12000 mg
Calcium D‐pantothenate	6000 mg
Vitamin B6	1200 mg
Vitamin B12	6000 mcg
Vitamin C	10,000 mg
Magnesium chloride	1000 mg
Zinc oxide	313 mg
Sodium iodate	6 mg
Iron chloride	294 mg
L‐Lysine HCl	432.5 mg
DL‐Methionine	94.5 mg
L‐Threonine	122.5 mg
Monosodium glutamate	500 mg
L‐Proline	310 mg
Glycine	257.5 mg
L Leucine	242.5 mg
L‐Valine	230 mg
L‐Arginine	202.5 mg
Aspartic acid	175 mg
L‐Alanine	162.5 mg
L‐Phenylalanine	150 mg
L‐Isoleucine	135 mg
L‐Tyrosine	81 mg
Maya Ekstresi	50,000 mg
Propylene glycol	20,000 mg

The injection was carried out at ED 12 since around that period, the embryo is developed enough to withstand any form of toxicity and stress. In addition, ED8‐12 is known as the most prevalent period of foetal myoblast availability, while ED5 is considered the most prevalent period of chick embryonic myoblast availability (Picard et al. 2002; Stockdale 1992). Other researchers (Piestun et al. 2015) also identified that ED7‐16 is the period when foetal myoblast activity occurs. Therefore, in ovo feeding at ED 12 could ensure maximum proliferation of cells, genes, and hormones involved in embryogenic growth and development. The amniotic sac was targeted as the site of injection due to the ease of application in that region, and also, the embryo has been reported to consume the amniotic fluid (Reicher et al. 2022; Ebeid et al. 2023). This indicates that injecting the supplement into the amniotic sac is a safe approach and also guarantees the utilization or absorption of the nutrient by the embryo.

At ED 18, candling was conducted, and fertile eggs were transferred to the hatcher with a temperature and humidity of 37.5°C and 70%, respectively.

### 2.2 Evaluation of Egg Weight, Egg Weight Loss, Embryonic Development, Hatchability, Embryonic Mortality, Chick Weight, Chick Yield, and Chick Quality

At embryonic day (ED) 16 and 19, all the eggs were weighed again using a scale with a precision of 0.1 g. The egg weight loss (EwL%) at different embryonic days was evaluated by subtracting the weight of the eggs at a specific ED from the weight of the eggs before incubation, dividing it by the weight of the eggs before incubation, and multiplying by 100%.

The embryonic morphological traits were evaluated at ED 16 and 19 using 5 eggs per group. The eggs were broken around the air space region (Agbehadzi et al. 2024), and embryos were killed by cervical dislocation (Ipek and Sozcu 2017). The embryonic traits assessed include YFBM‐w (weight of yolk‐free body mass) evaluated as the embryo weight without the yolk weight, W‐EY (embryo+yolk weight), Yolk‐w (yolk weight), Em‐W (embryo width), Em‐L (embryo length), Ey‐w (width of the eyes), TH‐L (thigh length), BL (beak length), WL (wing length), and TL (tibia length). A scale with 0.1 g precision was used to identify the Yolk‐w, YFBM‐w, and W‐EY. An INSIZE digital calliper (± 0.01 mm) was used to identify the BL, TH‐L, TL, Ey‐w, WL, Em‐W, and Em‐L. By dividing a certain embryo trait (Yolk‐w, YFBM‐w, W‐EY) by its egg weight and multiplying the result by 100%, the yield of the yolk‐free body mass (YFBM‐Y%), the yolk yield (Yolk‐y%), and the embryo+yolk yield (EY‐Y%) were identified. The embryonic features were measured in accordance with Kursun et al. (2025). Additionally, the traits were assessed using the following formula below (Kursun et al. 2025).

$$\begin{array}{ccc} \mathrm{EwL} & - & \mathrm{ED}\%\\ & = & \frac{\mathrm{Egg}\;\mathrm{weight}\;\mathrm{before}\;\mathrm{incubation}-\mathrm{Egg}\;\mathrm{weight}\;\mathrm{on}\;a\;\mathrm{specific}\;\mathrm{ED}}{\mathrm{Egg}\;\mathrm{weight}\;\mathrm{before}\;\mathrm{incubation}}\times 100 \end{array}$$

$$\begin{array}{ccc} & & \mathrm{Yield}\;\mathrm{of}\;a\;\mathrm{specific}\;\mathrm{embryo}\;\mathrm{trait}\;\left(\%\right)\\ & & =\frac{\mathrm{Weight}\;\mathrm{of}\;a\;\mathrm{specific}\;\mathrm{embryo}\;\mathrm{trait}}{\mathrm{Egg}\;\mathrm{weight}\;}\times 100 \end{array}$$

All chicks were weighed (CWAH) upon hatching using a 0.1 g precision scale, and all eggs that did not hatch were cracked to verify that the embryos had died. The following formula was used to assess the hatchability of set eggs (HSE), hatchability of fertile eggs (HFE), embryonic mortality (EM), and chick yield (CY). Thirty chicks from each treatment were used to assess specific chick quality traits (activity, eyes, legs, appearance, and navel) from the study by Tona et al. (2003). Using a ruler or rule set on a square table, the chicks’ length was measured from the beak to the middle toe claw (Kursun et al. 2025).

$$\;\mathrm{HSE}\%=\frac{\mathrm{Number}\;\mathrm{of}\;\mathrm{hatched}\;\mathrm{chicks}}{\mathrm{Total}\;\mathrm{number}\;\mathrm{of}\;\mathrm{eggs}\;\mathrm{set}}\times 100$$

$$\;\mathrm{HFE}\%=\frac{\mathrm{Number}\;\mathrm{of}\;\mathrm{hatched}\;\mathrm{chicks}}{\mathrm{Total}\;\mathrm{number}\;\mathrm{of}\;\mathrm{fertile}\;\mathrm{eggs}}\times 100.$$

$$\;\mathrm{EM}\%=\frac{\mathrm{Number}\;\mathrm{of}\;\mathrm{dead}\;\mathrm{embryos}}{\mathrm{Total}\;\mathrm{number}\;\mathrm{of}\;\mathrm{fertile}\;\mathrm{eggs}}\times 100.$$

$$\;\mathrm{CY}\%=\frac{\mathrm{Weight}\;\mathrm{of}\;\mathrm{chicks}\;\mathrm{at}\;\mathrm{hatch}}{\mathrm{Egg}\;\mathrm{weight}\;\;\mathrm{before}\;\mathrm{incubation}\;}\times 100.$$

### 2.3 Statistical Analysis

The normality test and test of homogeneity of the data were conducted using Shapiro‐Wilk and Levene's tests, respectively. It was confirmed that the actual and residual data showed a normal distribution assumption. After confirming the normality of the data, analysis of variance (ANOVA) was applied with fixed treatment effect (in ovo injection treatments) and replicate as a random effect. The *p*‐value was set at *p ≤* 0.05, and the confidence level/interval for the statistical analysis was 1 − 0.05 = 0.95, or 95%. The statistical software package JMP 18 (SAS 2017) was used for data analysis. All values are expressed as least squares means (LSMeans) ± pooled SEM (standard error of mean). The LSMeans were compared using the Tukey multiple comparisons test. The experimental effects consisted of T‐1 (non‐injected eggs), T‐2 (eggs injected with 5 mL of distilled water), T‐3 (eggs injected with 5 mL of a solution containing 0.04% of the dietary supplements), and T‐4 (eggs injected with 5 mL of a solution containing 0.08% of the dietary supplements). The statistical model is:

Yijk=μ+αi+eijk

μ=Overallmeancommontoallobservations

$$\alpha_{i}=\mathrm{Effect}\;\mathrm{of}\;\mathrm{in}\;\mathrm{ovo}\;\mathrm{treatments}$$

eijk=Randomerror

## 3 Results

The effect of in ovo injection of dietary supplements on egg weight and egg weight loss is shown in Table 2. The in ovo injection of dietary supplements had no effect on egg weight (EW) and egg weight loss (Ew‐L) at ED 19 (*p *> 0.05).

**TABLE 2:** The effect of in ovo injection of dietary supplement on egg weight and egg weight loss (mean ± SEM).

Groups	EW (g)		Ew‐L (%)
	EW‐BI	EW‐ED 19	Ew‐L‐ED 0‐19
T‐1	55.62 ± 0.32	50.02 ± 0.32	10.06 ± 0.59
T‐2	54.76 ± 0.33	50.04 ± 0.32	8.78 ± 0.59
T‐3	54.82 ± 0.32	50.44 ± 0.32	7.93 ± 0.59
T‐4	55.08 ± 0.32	50.48 ± 0.33	8.97 ± 0.59
*p*‐values	0.219	0.622	0.227

*Note*: T‐1 (non‐injected eggs), T‐2 (eggs injected with 5 mL of distilled water), EW (egg weight before incubation), T‐3 (eggs injected with 5 mL of a solution containing 0.04% of the dietary supplements), T‐4 (eggs injected with 5 mL of a solution containing 0.08% of the dietary supplements), SEM (standard error of mean).

The effect of in ovo injection of dietary supplements on embryonic traits at ED 16 is given in Table 3. The EW, W‐EY, EY‐Y, Yolk‐w, Yolk‐y, Ey‐w, TH‐L, and BL did not significantly differ among the experimental treatments (*p* > 0.05). The YFBM‐w, YFBM‐Y, TL, WL, Em‐W, and Em‐L were highest and lowest in T‐1 and T‐4, respectively (*p* < 0.05). The Em‐W, however, was highest in T‐3 and lowest in T‐4 (*p* < 0.05).

**TABLE 3:** The effect of in ovo injection of dietary supplement on embryonic traits at ED 16 (mean ± SEM).

Group	EW (g)	W‐EY (g)	EY‐Y (%)	YFBM‐w (g)	YFBM‐Y (%)	Yolk‐w (g)	Yolk‐y (%)	Em‐L (mm)	Em‐W (mm)	Ey‐w (mm)	TL (mm)	TH‐L (mm)	WL (mm)	BL (mm)
T‐1	50.32 ± 1.46	39.54 ± 1.72	78.54 ± 2.93	19.74 ± 0.39^a^	39.26 ± 0.89^a^	18.66 ± 1.51	37.06 ± 2.78	115.11 ± 1.46^a^	12.92 ± 0.43^a^	9.35 ± 0.32	18.03 ± 0.69^a^	13.02 ± 0.64	30.23 ± 0.75^a^	11.51 ± 0.38
T‐2	51.80 ± 1.46	36.22 ± 1.72	70.01 ± 2.93	17.98 ± 0.39^b^	34.75 ± 0.89^b^	17.58 ± 1.51	33.97 ± 2.78	107.65 ± 1.46^bc^	12.15 ± 0.43^ab^	8.92 ± 0.32	17.09 ± 0.69^a^	11.67 ± 0.64	29.73 ± 0.75^ab^	10.67 ± 0.38
T‐3	51.50 ± 1.46	34.60 ± 1.72	67.12 ± 2.93	18.00 ± 0.39^b^	35.06 ± 0.89^b^	15.78 ± 1.51	30.43 ± 2.78	109.24 ± 1.46^ab^	13.43 ± 0.43^a^	9.57 ± 0.32	15.70 ± 0.69^a^	11.63 ± 0.64	27.69 ± 0.75^ab^	11.38 ± 0.38
T‐4	50.80 ± 1.63	34.08 ± 1.92	74.62 ± 3.28	14.63 ± 0.43^c^	32.05 ± 1.00^b^	18.65 ± 1.69	40.89 ± 3.11	102.40 ± 1.63^c^	10.93 ± 0.48^b^	8.84 ± 0.36	12.31 ± 0.77^b^	10.46 ± 0.71	26.74 ± 0.84^b^	10.99 ± 0.43
*p*‐values	0.060	0.163	0.069	<0.001*	0.001*	0.526	0.119	<0.001*	0.008*	0.373	<0.001*	0.104	0.020*	0.425

*Note*: T‐1 (non‐injected eggs), T‐2 (eggs injected with 5 mL of distilled water), T‐3 (eggs injected with 5 mL of a solution containing 0.04% of the dietary supplements), T‐4 (eggs injected with 5 mL of a solution containing 0.08% of the dietary supplements), EW (egg weight), embryo weight without the yolk weight, W‐EY (embryo+yolk weight), Yolk‐w (yolk weight), Em‐W (embryo width), Em‐L (embryo length), Ey‐w (width of the eyes), TH‐L (tibia length), BL (beak length), WL (wing length), and TL (tibia length) SEM (standard error of mean), SEM (standard error of mean).

Different superscript letters “a, ab, b, bc, c” and “*” are significantly different (*p*≤0.05).

The effect of in ovo injection of dietary supplements on embryonic traits at ED 19 is given in Table 4. The EW, W‐EY, EY‐Y, Yolk‐w, Yolk‐y, TH‐L, YFBM‐w, YFBM‐Y, TL, WL, and BL did not significantly differ among the experimental treatments (*p >* 0.05). The Em‐L was highest in T‐3 and lowest in T‐4 (*p <* 0.05). In addition, the Ey‐w was highest in T‐3 and lowest in T‐1 (*p <* 0.05).

**TABLE 4:** The effect of in ovo injection of dietary supplement on embryonic traits at ED 19 (mean ± SEM).

Group	EW (g)	W‐EY (g)	EY‐Y (%)	YFBM‐w (g)	YFBM‐Y (%)	Yolk‐w (g)	Yolk‐y (%)	Em‐L (mm)	Em‐W (mm)	Ey‐w (mm)	TL (mm)	TH‐L (mm)	WL (mm)	BL (mm)
T‐1	49.32 ± 0.80	41.72 ± 0.97	84.60 ± 1.13	28.82 ± 0.55	58.52 ± 1.11	12.74 ± 0.64	25.75 ± 1.11	129.74 ± 2.28^ab^	14.20 ± 0.90	9.63 ± 0.40^b^	25.37 ± 0.65	17.64 ± 1.16	39.30 ± 1.48	15.56 ± 0.55
T‐2	49.22 ± 0.80	41.38 ± 0.97	84.06 ± 1.13	29.12 ± 0.55	59.16 ± 1.11	11.88 ± 0.64	24.15 ± 1.11	135.79 ± 2.04^a^	15.14 ± 0.90	9.76 ± 0.40^b^	26.34 ± 0.65	18.05 ± 1.16	39.40 ± 1.48	14.89 ± 0.55
T‐3	49.18 ± 0.89	42.63 ± 1.08	82.99 ± 1.46	27.85 ± 0.61	56.65 ± 1.24	12.87 ± 0.82	25.87 ± 1.39	136.60 ± 2.28^a^	14.91 ± 1.01	11.43 ± 0.46^a^	25.30 ± 0.65	19.40 ± 1.29	34.10 ± 1.66	13.97 ± 0.55
T‐4	49.64 ± 0.80	41.08 ± 0.97	82.75 ± 1.13	28.14 ± 0.55	56.72 ± 1.11	12.74 ± 0.64	25.66 ± 1.11	125.24 ± 2.04^b^	13.41 ± 0.90	10.19 ± 0.36^ab^	25.90 ± 0.65	16.85 ± 1.16	39.35 ± 1.48	15.72 ± 0.49
*p*‐values	0.977	0.745	0.659	0.392	0.328	0.705	0.669	0.006*	0.547	0.050*	0.646	0.541	0.087	0.137

*Note*: T‐1 (non‐injected eggs), T‐2 (eggs injected with 5 mL of distilled water), T‐3 (eggs injected with 5 mL of a solution containing 0.04% of the dietary supplements), T‐4 (eggs injected with 5 mL of a solution containing 0.08% of the dietary supplements), EW (egg weight), embryo weight without the yolk weight, W‐EY (embryo+yolk weight), Yolk‐w (yolk weight), Em‐W (embryo width), Em‐L (embryo length), Ey‐w (width of the eyes), TH‐L (tibia length), BL (beak length), WL (wing length), and TL (tibia length) SEM (standard error of mean).

Different superscript letters “a, ab, b, bc, c” and “*” are significantly different (*p*≤0.05).

The effect of in ovo injection of dietary supplements on hatching traits is shown in Table 5. The hatchbaility of set eggs (HSE) and hatchbaility of fertile eggs (HFE) were similar among the experimental treatments (*p >* 0.05). The embryonic mortality (EM) nearly reached a significant level, which was higher in T‐4 and lowest in T‐1 (*p =* 0.070). However, the chick weight at hatch (CWAH) and chick yield (CY) were higher in T‐4 (*p* < 0.05).

**TABLE 5:** The effect of in ovo injection of dietary supplement on hatching traits (mean ± SEM).

Group	HSE (%)	HFE (%)	EM (%)	CWAH (g)	CY (%)
T‐1	81.30 ± 4.70	82.38 ± 5.07	4.09 ± 3.59	39.29 ± 0.50^b^	70.07 ± 0.80^b^
T‐2	71.91 ± 4.70	74.83 ± 5.07	8.42 ± 3.59	39.91 ± 0.49^b^	71.65 ± 0.80^ab^
T‐3	67.96 ± 4.70	76.07 ± 5.07	7.49 ± 3.59	39.92 ± 0.50^ab^	72.34 ± 0.80^ab^
T‐4	58.61 ± 4.70	59.65 ± 5.07	22.81 ± 3.59	41.00 ± 0.50^a^	75.03 ± 0.80^a^
*p*‐values	0.107	0.123	0.070	0.046*	0.048*

*Note*: T‐1 (non‐injected eggs), T‐2 (eggs injected with 5 mL of distilled water), SEM (standard error of mean). T‐3 (eggs injected with 5 mL of a solution containing 0.04% of the dietary supplements), and T‐4 (eggs injected with 5 mL of a solution containing 0.08% of the dietary supplements).

Different superscript letters “a, ab, b, bc, c” and “*” are significantly different (*p*≤0.05).

The effect of in ovo injection of dietary supplements on chick quality traits is shown in Table 6. The chick length and appearance scores were lowest in T‐4; however, the eye score was lowest in T‐2 (*p <* 0.05). The activity, navel, and leg scores were similar among the experimental treatments (*p >* 0.05).

**TABLE 6:** The effect of in ovo injection of dietary supplement on chick quality traits (mean ± SEM).

Group	Chick length (cm)	Activity	Appearance	Eyes	Legs	Navel
T‐1	18.64 ± 0.08^a^	2.80 ± 0.55	9.27 ± 0.46^ab^	16.00 ± 0.23^a^	16.00	4.13 ± 0.99
T‐2	18.82 ± 0.08^a^	3.60 ± 0.55	9.93 ± 0.46^a^	15.20 ± 0.23^b^	16.00	6.13 ± 0.99
T‐3	18.30 ± 0.08^b^	3.20 ± 0.55	8.14 ± 0.47^bc^	16.00 ± 0.23^a^	16.00	4.55 ± 1.00
T‐4	18.22 ± 0.08^b^	3.20 ± 0.55	6.83 ± 0.47^c^	16.00 ± 0.23^a^	16.00	4.28 ± 1.00
*p*‐values	<0.001*	0.790	<0.001*	0.028*	—	0.457

*Note*: T‐1 (non‐injected eggs), T‐2 (eggs injected with 5 mL of distilled water), SEM (standard error of mean). T‐3 (eggs injected with 5 mL of a solution containing 0.04% of the dietary supplements), and T‐4 (eggs injected with 5 mL of a solution containing 0.08% of the dietary supplements).

Different superscript letters “a, ab, b, bc, c” and “*” are significantly different (*p*≤0.05).

## 4 Discussion

This study is the first to examine the in ovo injection of a single dietary supplement composed of vitamins, minerals, and amino acids in eggs, making it difficult to explain the findings; however, the lack of a significant effect of the dietary treatment on egg weight and egg weight loss at ED 19 could be associated with the inability of the experimental treatments to influence the eggshell conductance, the mechanism involved in facilitating the gaseous exchange between the developing embryo and its environment. The few studies that have investigated the in ovo injection of dietary supplements on egg weight have yielded different results. For instance, the findings of Ajayi et al. (2022) also revealed that in ovo injection of lysine, cystine, or their combination had no effect on egg weight. In addition, the studies of Mousstaaid et al. (2022) also revealed no effect of in ovo injection of L‐Ascorbic acid on egg weight loss during embryogenesis. However, the in ovo injection of L‐arginine has been reported to decrease egg weight loss (Taufik et al. 2023).

The YFBM‐w, YFBM‐Y, Em‐L, Em‐w, TL, and WL at ED 16 were significantly lower in T‐4, indicating that the in ovo injection of a mixture of dietary supplements (vitamins, minerals, and amino acids) at a concentration of 0.08% might have negatively influenced the cells, hormones, and genes involved in embryonic growth and development. Additionally, it is also possible that the in ovo injection of the supplement at a concentration of 0.08% might have increased the apoptotic death of embryonic cells as well as hindered the activation and expression levels of growth‐related genes (IGFBP2, GHRH, TRH, SST, TSHβ, and GH) in the hypothalamus, pituitary, and liver, resulting in poorer embryogenic growth and development. Moreover, it is possible that the in ovo injection of the supplement at a concentration of 0.08% might have decreased the expression of muscle growth factors (IGF‐1 and GH) and muscle marker genes (myogenin and MyoD) during embryogenesis, resulting in lower embryogenic growth and development. It is further speculated that the injection of the supplement at a concentration of 0.08% might have reduced the proliferation of the foetal and chick myoblasts through the deactivation of factors (Pax‐7, Shh, Wnt‐3, and PCNA) that control myogenesis, resulting in decreased embryogenic muscle size and the number of myoblasts. Indeed, lower mRNA levels of IGF‐I and IGF‐II in embryos injected with different amounts of minerals (Zn) have been reported by Jadhav (2025), while Bölükbaş and Öznurlu (2023) also identified a higher number of necrotic neurons in embryos injected with different levels of monosodium glutamate. The findings of the present study also confirm the results of Ohta et al. (2001) and Kop–Bozbay Ocak (2019), who also reported a lower embryonic weight in eggs injected with amino acids. In addition, the injection of higher amounts of monosodium glutamate has been associated with poorer eye development (reduction in corneal epithelium thickness, total corneal thickness, and ganglion cell number) in embryos (Bölükbaş and Öznurlu 2023) at ED 15, 18, and 21. However, a higher embryonic weight in eggs injected with amino acids has been reported (Kita et al. 2015). Taufik et al. (2023) also reported that although the embryo weight and yield were higher in eggs injected with L‐arginine at ED 16, the embryo length, wing length, and yolk weight were similar among the experimental treatments.

The higher embryonic length in T‐3 embryos at ED 19 suggests that the in ovo injection of the dietary supplement at a concentration of 0.04% might have enhanced the stimulation, activation, and proliferation of cells (osteoclasts) and bone morphogenetic proteins (BMP8A, SOX9, and RUNX2), which are responsible and crucial for chondrocyte functionality and bone development. Indeed, embryos injected with L‐arginine had been characterized by a higher embryonic length at ED 18 (Azhar et al. 2016). In addition, the improved eye width in T‐3 embryos at ED 19 also indicates that the injection of the dietary supplement at a concentration of 0.04% improved the corneal epithelium thickness, total corneal thickness, and ganglion cell number in embryos, resulting in better eye development at ED 19.

The chick weight and chick yield were significantly higher in T‐4 compared with the other experimental treatments, and it is speculated that the injection of the dietary supplement at a concentration of 0.08% improved the utilization of yolk, resulting in better embryonic growth during the late stage of embryogenesis. Moreover, it could be possible that the injection of the dietary supplement at a concentration of 0.08% enhanced the proliferation of growth cells (chick and foetal myoblast/satellite cells) and genes (IGF‐I, IGF‐II, etc.) during late embryogenesis, resulting in an improved embryonic development with a subsequently enhanced chick weight and chick yield at hatch. Indeed, a higher chick weight at hatch in eggs injected with zinc, iron, and copper sulphate has been identified by Elokil et al. (2021). The in ovo injection of organic minerals at a high concentration has been identified with an improvement in chick weight at hatch (Oliveira et al. 2015). In addition, some studies (Ohta et al. 2001; Kop‐Bozbay Ocak 2019; Ajayi et al. 2022) that have investigated the effect of in ovo injection of amino acids in eggs have also reported a higher chick weight and chick yield at hatch in eggs injected with a single or a combination of different dietary amino acids. Moreover, an improvement in chick weight and chick yield at hatch in eggs injected with vitamins has been reported by Araújo et al. (2019) and Kalaba et al. (2024). However, different studies (Salmanzadeh et al. 2012; Sahr et al. 2020) have reported lower chick weight at hatch in eggs injected with minerals compared with chicks from the control treatment.

The hatchability traits and embryonic mortality were similar among the experimental treatments, and it was speculated that the injection of the dietary supplement did not influence the cells, hormones, and genes (thyroid hormones, especially thyroxine, T‐4, caspase‐3) involved in the hatching processes of chicks or involved in causing the apoptotic death of developing embryos. Comparing our findings to studies that have investigated the in ovo injection of a single dietary nutrient, the in ovo injection of zinc, iron, and copper sulphate has been reported to have no effect on hatchability and early embryonic mortality (Elokil et al. 2021). Eggs injected with 40 µg of vitamin B12 at ED 15 have also been reported to have similar hatchability values compared with eggs that served as the control treatment (Teymouri et al. 2020). In addition, Kalaba et al. (2024) also confirmed that the injection of Vitamin E, B1, and B2 had no effect on hatchability and embryonic mortality. However, some studies on amino acids have reported either improved or reduced hatchability (Kop‐Bozbay Ocak 2019; Taufik et al. 2023) due to the in ovo injection of amino acids in eggs.

In the present study chick length was lowest in the T‐4 and it was speculated that the injection of the supplement at a concentration of 0.08% might have decreased the proliferation, stimulation, and activation of the cells (osteocytes and osteoblasts) and genes (SDC3, BMP8A, COL10A1, BMP8A, and SCIN, TGF‐β1) involved in the growth and development of the skeletal system (Abdallah et al. 2026) during the latter stages of incubation (ED 20‐21). In addition, Makarova et al. (2019) reported that plumage colouration results from two distinct but connected physical processes: (a) the chemical mechanism, which produces colouration due to substances that absorb a specific wavelength and form so‐called pigment colours; and (b) the optical mechanism, which produces structural colours due to interference of light reflected by the biological microstructures of the feathers. Therefore, it is hypothesized that the injection of the supplement at a concentration of 0.08% might have caused significant changes in the mechanism or pigmentation processes of the plumage, resulting in poor feather development, colouration, and pigmentation of the feathers during embryogenesis, with a subsequent poorer appearance score at hatch in T‐4 chicks. Indeed, eggs injected with a dietary solution containing a mixture of 1.7 mg cysteine and 1 mg lysine have been identified with the poorest chick quality score at hatch (Ajayi et al. 2022). The in ovo injection of certain concentrations of vitamin E (27.5 and 38.5 IU) has also been reported to decrease chick quality score at hatch (Araújo et al. 2019).

The eye score was lowest in T‐2 chicks, which indicates that the injection of distilled water in eggs might have decreased the corneal epithelium thickness, total corneal thickness, and ganglion cell number during embryogenesis, resulting in poorer eye development or sensitivity at hatch. Moreover, the activity of chicks, leg, and navel score did not vary among the experimental treatments, which agrees with the findings of Ajayi et al. (2022), who also reported that eggs injected with amino acids did not differ in terms of chick activity, leg, and navel score at hatch.

It was therefore concluded that the in ovo injection of a complex nutritional solution may improve some aspects of embryonic development during the latter stages of embryogenesis, chick weight, and chick yield at hatch; however, it may possess a detrimental effect on embryonic mortality, hatchability, and chick quality.

## Author Contributions

**Kadriye Kursun**: conceptualization, investigation, writing – original draft, writing – review and editing, visualization, validation, methodology, software, formal analysis, project administration, supervision. **Nasir Abdallah**: conceptualization, investigation, writing – original draft, writing – review and editing, visualization, validation, methodology, software, data curation, formal analysis, project administration, supervision. **Mikail Baylan**: investigation, project administration.

## Funding

The authors have nothing to report.

## Ethics Statement

This study was conducted under the guidelines for animal experiments of the Ministry of Food, Agriculture and Livestock, Türkiye. Approval was granted by the animal experiments local ethics committee of Cukurova University.

## Conflicts of Interest

The authors declare no conflicts of interest.

## Statement of AI

No AI was used in the preparation of this study.

## Data Availability

The data for this study are available from the corresponding author and will be shared upon request.
